# Automated cone photoreceptor detection using synthetic data and deep learning in confocal adaptive optics scanning laser ophthalmoscope images

**DOI:** 10.1038/s41598-026-39570-9

**Published:** 2026-02-11

**Authors:** Mital Shah, Laura K. Young, Susan M. Downes, Hannah E. Smithson, Ana I. L. Namburete

**Affiliations:** 1 Oxford Eye Hospital, John Radcliffe Hospital, Oxford University Hospitals NHS Foundation Trust, Oxford, OX3 9DU UK; 2 Nuffield Laboratory of Ophthalmology, Nuffield Department of Clinical Neurosciences, University of Oxford, Level 6 John Radcliffe Hospital, Headley Way, Oxford, OX3 9DU UK; 3 Department of Experimental Psychology, University of Oxford, Oxford, OX2 6GG UK; 4 Biosciences Institute, Newcastle University, Newcastle, NE2 4HH UK; 5 Oxford Machine Learning in NeuroImaging (OMNI) Laboratory, Department of Computer Science, University of Oxford, Oxford, OX1 3QG UK

**Keywords:** Adaptive optics scanning laser ophthalmoscope, Cone photoreceptor, Deep learning, Artificial intelligence, Convolutional neural network, Synthetic data, Medical research, Mathematics and computing

## Abstract

Adaptive optics scanning laser ophthalmoscope (AOSLO) imaging enables the cone photoreceptor mosaic to be visualised in the living human eye. Performing quantitative analysis of these images requires identification of individual photoreceptors. This is typically performed by manual labelling, which is subjective, time consuming and not feasible on a large scale. Automated algorithms to replace manual labelling are required and deep learning-based methods provide an effective way of achieving this. However, this approach requires large volumes of annotated training data that are difficult to acquire. Synthetic data may help to bridge this lack of annotated training data. A U-Net configuration was trained using a large synthetic dataset of confocal AOSLO images generated using ERICA alongside a smaller dataset of real confocal AOSLO images (Milwaukee dataset). Model performance was assessed by calculating the Dice coefficient, a metric quantifying segmentation overlap, on both a real held-out test set and an independent real dataset (Oxford dataset). Results from this evaluation were benchmarked against expert labelling and two automated cone detection methods: a confocal convolutional neural network (CNN) (1), and a combined graph-theory and dynamic programming approach (2)). The mean Dice coefficient compared to manual labelling was 0.989 (U-Net), 0.989 (confocal CNN), and 0.985 (graph-theory and dynamic programming) on the held-out test set. On the independent Oxford dataset, the U-Net achieved a mean Dice coefficient of 0.962 compared to manual labelling. Results show performance that is comparable to the gold standard of manual labelling and two automated cone detection methods. Furthermore, we demonstrate generalisability of this approach on an independent real dataset with images from higher retinal eccentricities. This approach may be useful for quantitative analysis of the photoreceptor mosaic in patients with retinal disease to provide cell-specific imaging biomarkers from AOSLO images.

## Introduction

Adaptive optics scanning laser ophthalmoscopy (AOSLO) enables individual photoreceptors, including foveal cones and peripheral rods, to be visualised in the living human eye. The cellular resolution achieved with AOSLO imaging provides the ability to quantify changes in the living photoreceptor mosaic. This is useful for providing diagnostic and prognostic information about retinal diseases, for understanding disease mechanisms, and for evaluating novel treatments with cell-specific imaging biomarkers. Identification of individual photoreceptors is often a prerequisite for generating quantitative metrics of the photoreceptor mosaic (such as photoreceptor density or reflectivity). Manual photoreceptor identification is both time consuming and subjective, potentially leading to inaccurate estimates of these metrics.

A number of algorithmic approaches have been developed for automated cone detection in adaptive optics images^2–6^. However, while they may show good performance for the specific problem for which they were designed, their reliance on a predefined set of rules limits their generalisability. For example, the adaptive filtering and local detection method described by Cunefare et al.^7^, which was developed using images from healthy volunteers, performed poorly when detecting cones from images of participants with Stargardt disease^8^. Many algorithms developed for automated cone detection still require manual supervision when detecting cones in diseased eyes or in low-quality images^9^.

Deep learning provides an effective way of automatically extracting and learning features directly from the image data, potentially increasing generalisability. Traditional deep learning approaches require large volumes of training data. Acquiring large annotated datasets of the photoreceptor mosaic from confocal AOSLO images in healthy volunteers is difficult. Furthermore, gathering datasets that are representative of the patient cohorts seen in real-world healthcare settings is even more challenging. These datasets also require accompanying annotations generated by experts, which are time consuming and expensive to produce.

An alternative is to use synthetic AOSLO data, allowing generation of large quantities of training images with known ground truth labels. The aim of this study was to investigate if synthetic data could be used to fit a novel convolutional neural network (CNN) variant to automatically identify cone photoreceptors from in vivo confocal AOSLO images.

## Methods

**Fig. 1:**
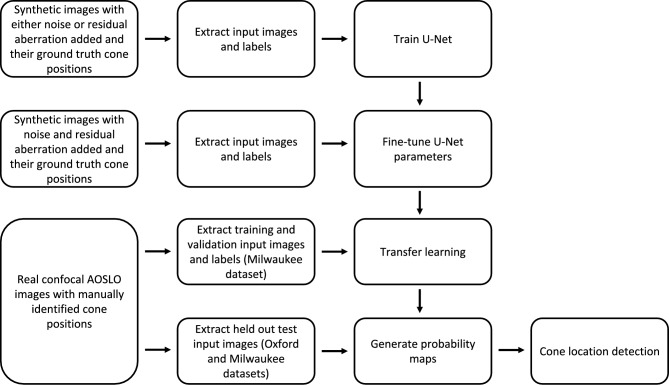
Summary of U-Net based approach for cone detection.

The U-Net is a CNN architecture originally developed for biomedical image segmentation^10^, characterised by a symmetric encoder-decoder structure. In this study, we adapted the U-Net for cone detection in confocal AOSLO images, as summarised in Fig. 1. Initially, the model was trained on a synthetic dataset simulating the effects of either noise or residual optical aberrations (details below). Fine-tuning was then performed using a second synthetic dataset that incorporated both factors simultaneously. To enhance the model’s performance on real-world data, we applied transfer learning by initialising the network with parameters (weights and biases) learned from synthetic data and then fine-tuning it using real AOSLO images. To detect cone locations at inference time, the fine-tuned U-Net was applied to previously unseen real confocal AOSLO images to produce probability maps. Cone detection was achieved by applying a threshold to the probability maps prior to using a peak local maximum function to extract individual cone locations.

### Synthetic data

**Fig. 2:**
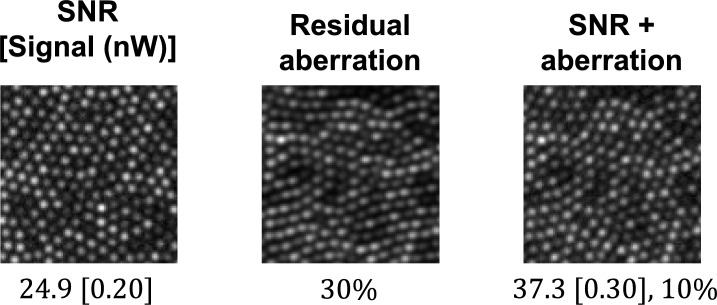
Examples of the averaged synthetic input images (from 5°) used for training the U-Net. Left – Example synthetic input image (generated by averaging 20 single synthetic frames) that included noise and the effects of diffraction. The image is labelled with its signal to noise ratio (SNR) and the image signal in parentheses, quantified in nanowatts (nW) at the pinhole. The image signal was varied from 0.20nW to 0.35nW. Middle – Example synthetic input image that included residual aberration. An average over the time series is used as the Zernike coefficient to apply. The percentage of the wavefront amplitude used to simulate residual aberration was varied from 10 to 30%. Right – Example synthetic input image that included the interaction between noise and residual aberration. An image signal of 0.3 nW was used and 10% of wavefront amplitude applied to simulate residual aberration.

We used a previously reported simulation tool, ERICA (Emulated Retinal Image CApture), that generates synthetic images of the human cone photoreceptor mosaic^11^. ERICA mimics confocal AOSLO data capture, including effects such as aberrations and noise, and outputs realistic *en face* images of the cone photoreceptor mosaic, with corresponding ground-truth data. Each generated synthetic image contained a different pattern of reflectivity across photoreceptors, to mimic different retinae. Synthetic images providing a realistic cone photoreceptor mosaic were generated to represent 19 retinal eccentricities (1° to 10° in 0.5° steps), with a pixel scale of one micron. These images were 480 × 300 pixels in size and 100 images were generated for each retinal eccentricity. The images did not simulate rod photoreceptors or retinal blood vessels. To accurately represent real AOSLO images three sets of synthetic images were produced, each of which included the effects of diffraction (imaging wavelength of 850 nm and a 5mm pupil diameter), with: i) added noise, ii) added residual aberrations, and iii) a combination of added noise and residual aberration (Fig. 2).

#### Added noise

An important measure of confocal AOSLO image quality is the signal-to-noise ratio (SNR) and the SNR was altered in simulation by varying the intensity signal (measured in nanowatts) at the pinhole. By considering noise introduced at any point in the system as constant in its characteristics over time, randomly generated Gaussian distributed white noise was added to the synthetic images. The parameters of this Gaussian distribution were matched to those measured from images captured with an AOSLO^12^. The time-average simulated power at the pinhole was determined heuristically and varied from 0.2 nanowatts (nW) to 0.35 nW in steps of 0.05 nW, to simulate increasing corneal exposures (or increasing retinal reflectivity) while imaging. Real AOSLO images are normally acquired by registering and averaging multiple single raw frames. In our simulation, averaged images were produced by averaging 10, 20 or 30 single frames but registration was not required since eye movements were not included.

#### Added residual aberrations

We used time-series measurements of wavefront aberrations, quantified by Zernike coefficients, from healthy eyes obtained by Jarosz et al.^13^. The mean Zernike coefficient for each measured mode was calculated across the time sequence for each eye. We then selected four eyes with the lowest total RMS amplitude of wavefront error for further use. For each of the four eyes a wavefront was reconstructed from its Zernike coefficients but with a fixed proportion (30%, 20% or 10%; determined heuristically) of the amplitude. The point spread function corresponding to the scaled wavefront was then used to introduce residual aberration in the rendering of the synthetic images via convolution^11^.

#### Added noise and residual aberration

Zernike coefficients from 10 eyes from the Jarosz et al. dataset^13^ with the lowest total RMS amplitude of wavefront error were used. Residual aberrations were added through convolution, as described above, using a wavefront with 10% of the amplitude. Following this, Gaussian distributed white noise was added, the time-average signal strength was set to 0.3 nW at the pinhole, and images were generated by averaging 20 single frames.

### Milwaukee dataset

**Fig. 3:**
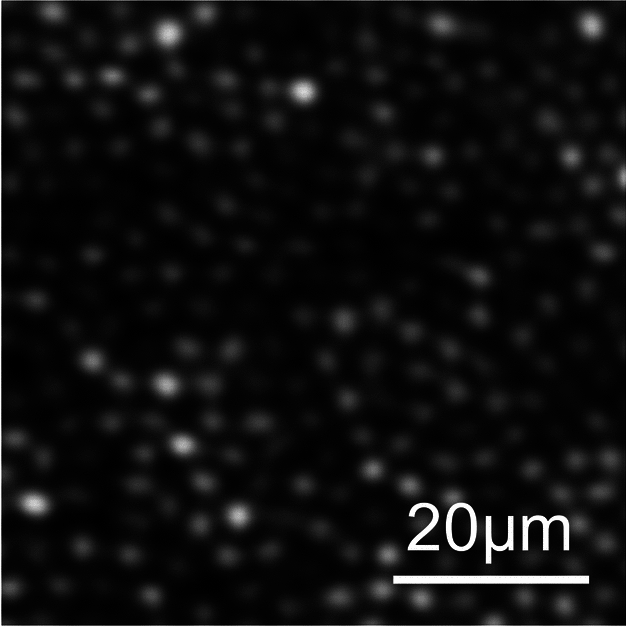
Example confocal AOSLO image from the Milwaukee dataset at 0.65° from the fovea.

This publicly available dataset of 840 confocal AOSLO images from 21 participants (20 normal, one deutranopic) has been previously used for the development and validation of cone photoreceptor segmentation and identification algorithms^2,7^. Four retinal locations 0.65° from fixation were imaged for each participant (supero-temporal, supero-nasal, infero-nasal, infero-temporal) and 10 averaged images were acquired per location (Fig. 3). The size of each 150 × 150 pixel image on the retina ranged from 58 µm^2^ to 71 µm^2^. All images were accompanied by semi-automatically marked annotations of the cone photoreceptors performed by an expert grader. Training and validation data were augmented by 200 fold using rotation and flipping along the x-axis. The held-out test set included 160 images from four participants that were included in the validation data set used by Cunefare et al.^1^ for evaluating model performance.

### Oxford dataset

**Fig. 4:**
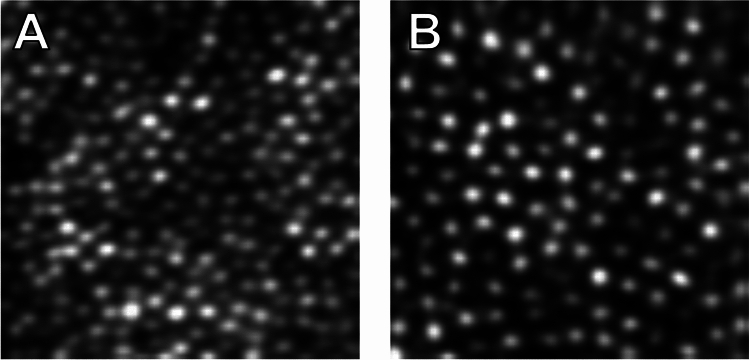
Example confocal AOSLO images from the Oxford dataset of one participant. **A**—3° temporal to the fovea, **B**—6° temporal to the fovea.

To extend our data beyond the fovea to assess generalisability of the algorithms, we used additional data collected in-house. This dataset consisted of a total of 17 images 100 × 100 pixels in size from 7 normal participants. All experimental protocols were approved by the Medical Sciences Interdivisional Research Ethics Committee (reference R58464/RE001). The study was conducted in accordance with relevant guidelines and regulations, and informed consent was obtained from all subjects. Images were obtained from a previously described confocal AOSLO^12^ with a 1.0° × 1.6° field of view. Two eccentricities (3° and 6°) in the temporal and superior meridians from fixation were imaged and sufficient data were collected to produce one averaged image per location (Fig. 4). Cones were manually identified in all images by an experienced grader. The validation dataset included four images from one participant. The test set included the remaining 13 images from six participants.

### Input images

All input images were cropped to 96 × 96 pixels. To ensure balanced representation of synthetic data between training, validation, and testing sets, the datasets were stratified according to image characteristics such as added noise, residual aberrations, and retinal eccentricity. Synthetic images were split into 12 non-overlapping sub-images, resulting in a total of 775,200 input images. The Milwaukee dataset was split into training (60%), validation (20%), and testing (20%) sets. To avoid data leakage with real data, images acquired from the same participant at the same retinal eccentricity were kept within a single dataset split.

### Training labels

**Fig. 5:**
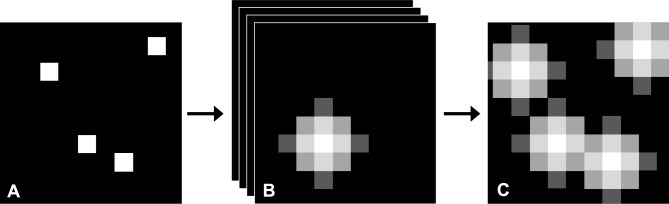
Ground truth Gaussian annotated training labels. **A**—map of the ground truth cone centres where each cone centre is represented by a single pixel. **B**—each pixel representing a cone centre from the map of cone centre locations is separated (while maintaining its spatial position in the XY plane) to create a Z stack and these were subsequently convolved with a Gaussian kernel. **C**—Maximum intensity projection is used through the Z stack onto a 2D image to eliminate the problem of overlapping Gaussians.

Ground-truth labels were created by convolving maps of known cone centres (defined in the simulation for synthetic data and specified by manual labels for real data) with a Gaussian kernel to create smooth training annotations (Fig. 5). Gaussian annotations were truncated to the diameter of a single cone to minimise overlap, and maximum intensity projection was applied to resolve the problem of overlapping Gaussians. Final training label intensities were normalised to the [0, 255] range.

### Convolutional neural network

The model was implemented in Python using the Keras API^14^ with a TensorFlow backend^15^. Training was conducted on an NVIDIA Quadro P4000 graphics processing unit (GPU) for synthetic data and an NVIDIA Tesla T4 GPU for transfer learning.

**Fig. 6:**
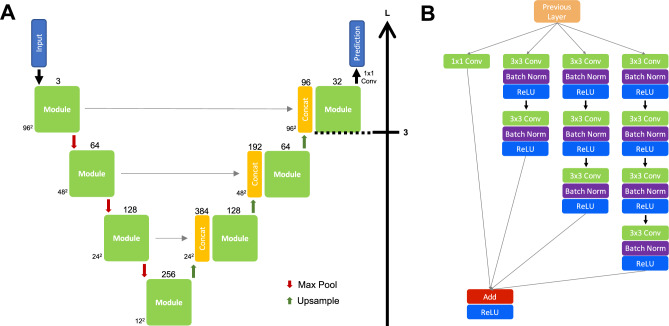
The U-Net architecture. Inputs and predictions are 96 × 96 pixels in size. **A**—Overview of the U-Net architecture. The green boxes represent the module architecture shown in B. The input size in pixels and the number of filters are provided at the lower left edge and on top of each box, respectively. The horizontal grey arrows represent higher resolution feature maps from the contracting path that are concatenated with upsampled features from the expansive path. The max pooling window is set to 2 × 2 with a stride of 2. L—layer depth for transfer learning. **B**—The module architecture illustrated by the green boxes in A. Add—matrix addition, Batch Norm—batch normalisation, Concat—concatenation, Conv—convolutional layer (with kernel size specified), Max Pool—maximum pooling, ReLU—Rectified Linear Unit.

Variability in cone width across retinal eccentricities and the effect of differing image resolutions between instruments poses a challenge for consistent detection. Specifically, cone width (in microns) increases with retinal eccentricity, and so the measured cone width (in pixels) also increases. However, the cone width in pixels will also increase for images with a smaller pixel scale (in microns). To address this, we designed a modified U-Net architecture capable of capturing multi-scale features (Fig. 6). Our architecture extends the standard U-Net design by incorporating parallel convolutional layers with multiple kernel sizes at each level, enabling more explicit multi-scale feature extraction. This design draws inspiration from Inception networks^16^ and enables the model to better capture structural variations across the retina. Additionally, residual shortcut connections^17^ were included to facilitate gradient flow and mitigate vanishing gradients.

The final model contained approximately 8.9 million trainable parameters. Mean squared error was used as a loss function, with weights initialised using a random orthogonal matrix. Optimisation was performed using Adam^18^ with an initial learning rate of 10^–3^, which was reduced by a factor of two if no improvement in validation loss ($\ge$ 1) was observed after five epochs, down to a minimum of 10^–5^. For transfer learning, the initial learning rate was 10^–4^, with similar decay criteria. During fine-tuning, only the last convolutional block (L3) on the expansive path (Fig. 6) was unfrozen for training.

All convolutional layers used a 3 × 3 kernel with zero padding to preserve spatial dimensions. Batch normalisation was applied prior to the activation functions. Training was conducted with a batch size of 32 and four-fold cross-validation. Early stopping was triggered if no reduction in validation loss $\ge$ 1 was observed over seven consecutive epochs.

### Evaluation protocol

The model was evaluated on two real datasets, the held-out test set from the Oxford and Milwaukee datasets. The number of true positives (TP), false positives (FP) and false negatives (FN) were calculated for each test image. Dice coefficient, true positive rate, and false discovery rate were used to evaluate the accuracy of the model and were defined as:

$$\text{Dice coefficient }=2TP/ (2TP+FP+FN)$$

$$\text{True positive rate}= TP/(TP+FN)$$

$$\text{False discovery rate}=FP/(TP+FP)$$

**Fig. 7:**
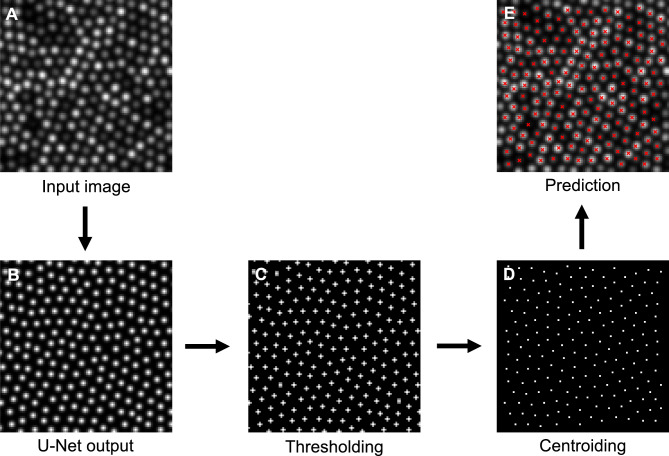
Identification of cone centres from the predicted cone probability map. **A**—the input image to the U-Net. **B**—the predicted cone location output by the U-Net where intensity represents the probability of a cone. **C**—the cone probability map after a minimum threshold (*T*) has been applied. **D**—the centroids of all predicted cones after thresholding (each peak represents the centre of a predicted cone). **E**—an overlay of D on A illustrating the centre of each predicted cone with a red cross. To avoid misinterpretation of the binary mask shape, cone locations are extracted from the continuous probability map using a probability-weighted centroid; binary masks shown in this figure are for visualisation only and result from hard thresholding of the probability map.

**Fig. 8:**
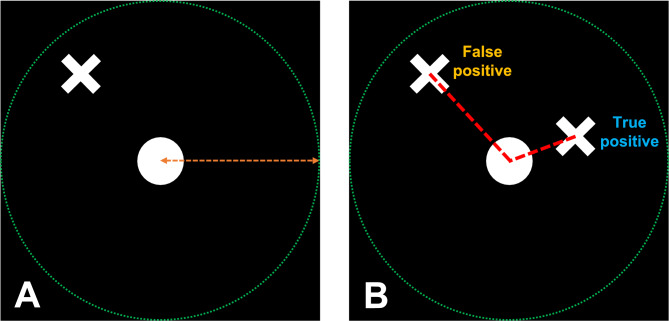
Identifying true and false positive predicted cones. **A**—if a predicted cone centre (white cross) is within χ cone widths (orange dotted arrow) of a ground truth cone centre (white circle), a cone pair is formed. **B**—if the centre of more than one predicted cone (white cross) can form a cone pair with a ground truth cone centre (white circle), the predicted cone with the shortest Euclidean distance (dotted red line) is paired with the ground truth cone and classified as a true positive while the other predicted cone is classified as a false positive. Green dotted circle represents the circumference of a ground truth cone based on the cone width.

Individual cone locations were detected by processing the predicted cone probability maps. Briefly, cone centres were identified from the predicted cone probability maps by applying a minimum intensity threshold *T* and identifying the centroids of all peaks using a peak local maximum function (Fig. 7). Predicted cone centres were considered true positive if their distance to the nearest ground truth cone centre was a fraction χ of a cone width (calculated from the input image for the predicted cone probability map being evaluated). Each ground truth cone could only be paired with one predicted cone (shortest Euclidean distance, Fig. 8). Cones within distance χ cone widths of the edge of the input image or the edge of the predicted cone probability map were excluded from evaluation. The intensity threshold *T* and distance χ were determined heuristically using the validation datasets by maximising the Dice coefficient when compared to manual labelling. The values *T* and χ were determined as 45 and 0.5 for the Oxford dataset, and 120 and 0.75 for Milwaukee dataset.

### Comparison with state-of-the-art

We evaluated the model against the current gold standard of manual annotation with the Oxford and Milwaukee datasets. Additionally, we compared our model with two alternative automated methods of cone detection, a confocal CNN (C-CNN)^1^ and graph-theory and dynamic programming (GTDP)^2^. The C-CNN and GTDP methods were implemented using the code published by Cunefare et al.^1^ and were evaluated on the Milwaukee held-out test set described in this study.

## Results

Training was conducted in three stages. First, the synthetic data including noise or residual aberration were used to fit the model’s parameters, which took 60 epochs. Parameter fine-tuning was then carried out using synthetic data that considered the interaction between noise and residual aberration, which took 10 epochs. Finally, transfer learning was used with the Milwaukee dataset, which took 37 epochs.

**Fig. 9:**
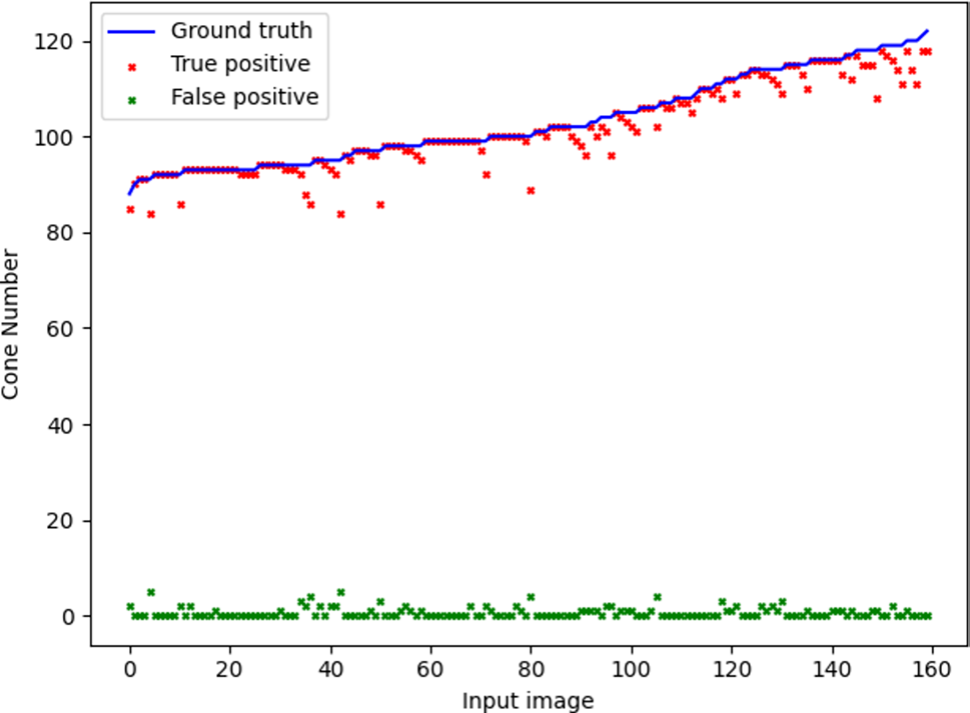
Performance of the U-Net compared to manual labelling on the Milwaukee dataset. Results from all images in the Milwaukee held-out test set are included. The solid blue line represents the true number of cones from manual labelling for each input image. Red crosses represent the number of true positive cones for each image. Green crosses represent the number of false positive cones for each image. The number of false negative cones are represented by the difference between the number of true cones (blue line) and true positive cones (red cross).

**Fig. 10:**
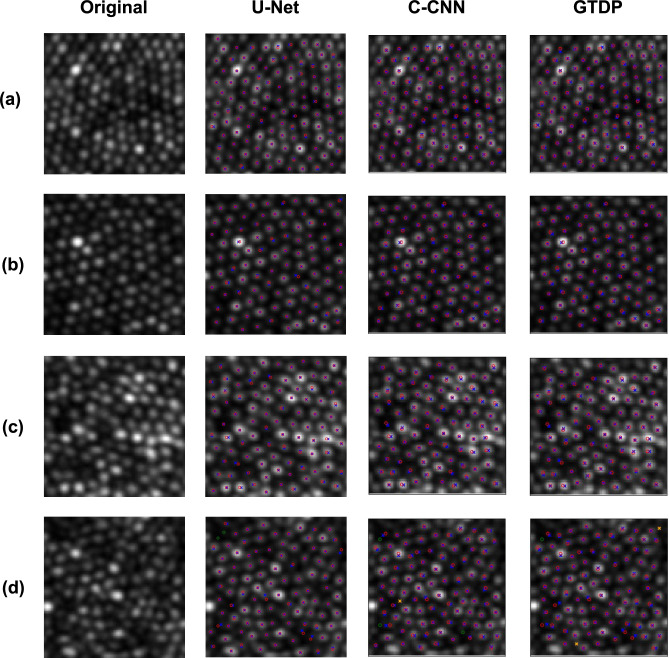
Performance of the U-Net and two alternative automated methods of cone detection compared to manual labelling on real data from the Milwaukee dataset. One image for each participant in the held-out test dataset has been included. Crosses denote predicted cone centre locations with blue crosses denoting true positives and orange crosses denoting false positives. Circles denote ground truth cone centre locations with green circles denoting misses (false negatives) and red circles denoting hits (true positives). C-CNN—confocal convolutional neural network^1^ for cone detection, GTDP—graph-theory and dynamic programming^2^ cone detection method.

The average performance of the U-Net, C-CNN and GTDP cone detection methods on the Milwaukee held-out test set are summarised in Table 1. The mean Dice coefficient was 0.989 for the U-Net, 0.989 for the C-CNN and 0.985 for the GTDP methods. Results from all images in the Milwaukee held-out test set showing the number of true positive, false positive, and false negative cones are displayed in Fig. 9. Figure 10 displays the results of all three cone detection methods for four example images from the Milwaukee held-out test set compared to manual labelling.

**Table 1:** Results of the U-Net and two alternative automated methods of cone detection.

	True positive rate (SD)	False discovery rate (SD)	Dice coefficient (SD)
U-Net	0.985 (0.024)	0.006 (0.011)	0.989 (0.016)
C-CNN*	0.988 (0.015)	0.010 (0.014)	0.989 (0.013)
GTDP*	0.988 (0.016)	0.018 (0.020)	0.985 (0.016)

C-CNN—confocal convolutional neural network^1^, GTDP—graph-theory and dynamic programming^2^, SD—standard deviation, *these cone detection methods were implemented using the code published by Cunefare et al.^1^ and evaluated on the Milwaukee held-out test set (160 images from four participants) described in this study.

**Fig. 11:**
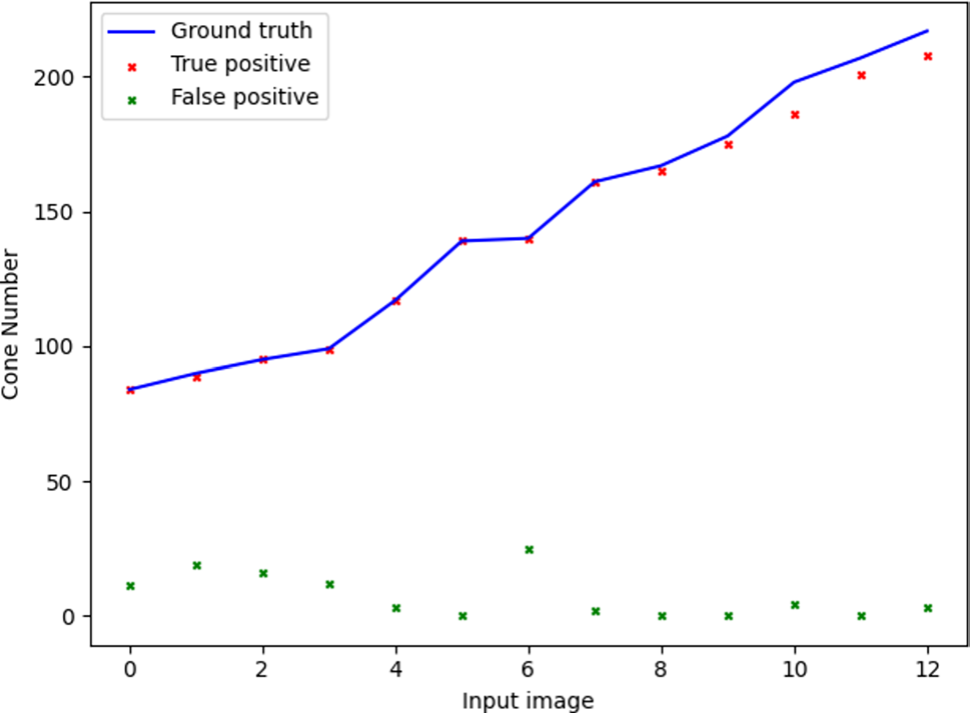
Performance of the U-Net compared to manual labelling on the Oxford dataset. Results from all images in the Oxford held-out test set are included. The solid blue line represents the true number of cones from manual labelling for each input image. Red crosses represent the number of true positive cones for each image. Green crosses represent the number of false positive cones for each image. The number of false negative cones are represented by the difference between the number of true cones (blue line) and true positive cones (red cross).

**Fig. 12:**
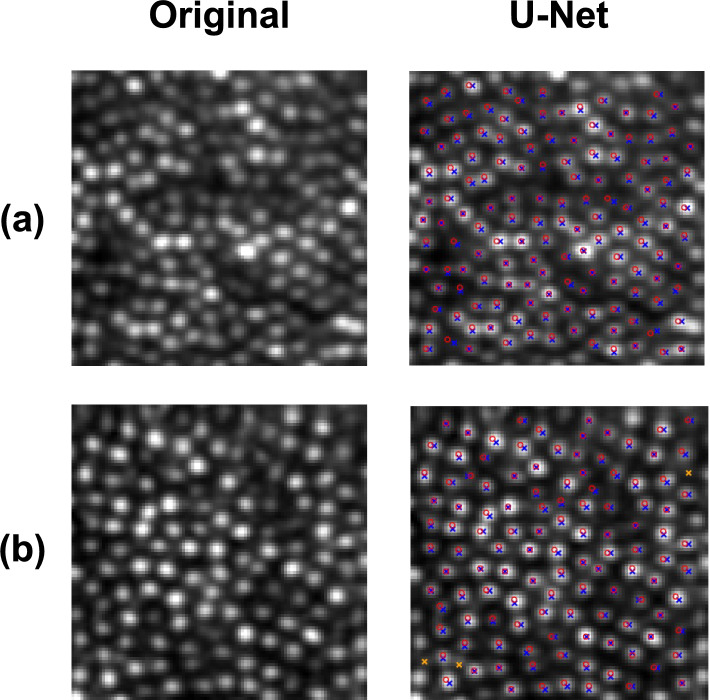
U-Net performance compared to manual labelling on real data from the Oxford dataset. Two images from one participant at 3° from fixation in the superior meridian (**a**) and 6° from fixation in the temporal meridian (**b**) are illustrated. Crosses denote predicted cone centre locations with blue crosses denoting true positives and orange crosses denoting false positives. Circles denote ground truth cone centre locations with green circles denoting misses (false negatives) and red circles denoting hits (true positives).

Evaluating the U-Net on the Oxford test set gave a mean Dice coefficient of 0.962 (SD = 0.033), mean true positive rate of 0.987 (SD = 0.019), and mean false discovery rate of 0.059 (SD = 0.066). Results from all images in the Oxford test set showing the number of true positive, false positive, and false negative cones are displayed in Fig. 11. Figure 12 displays the results of the U-Net compared to manual labelling for two example images from the Oxford test set.

## Discussion

This study demonstrates that a fully automated deep learning-based method for identifying cone photoreceptors in confocal AOSLO images, which was trained using synthetic data, has a good agreement with the current gold standard of manual marking. Using synthetic images of the cone photoreceptor mosaic over a wide range of retinal eccentricities provided sufficient data to train a newly developed CNN and leverage the learned feature maps with manually marked real images to identify cone photoreceptors. The success of this approach demonstrates the suitability of using synthetic data to overcome the limited availability of annotated real datasets. The use of synthetic data over a wide range of retinal eccentricities also increases the generalisability of a trained model to confocal AOSLO images taken over a similarly wide range of retinal eccentricities.

Evaluating the proposed U-Net on the Milwaukee dataset yielded a Dice coefficient of 0.989 (Table 1). These results compare favourably with manual marking, considered to be the current gold standard, and with two alternative automated methods of cone detection^1,2^ that have been evaluated using the same dataset^1^. Although our results are promising, it is important to note that we evaluated our algorithm using 160 images from four participants in the Milwaukee dataset. Cunefare et al. evaluated the automated algorithms using 640 images from 16 participants in the same dataset and yielded a Dice coefficient of 0.990 for the C-CNN and 0.988 for the GTDP method^1^. Evaluating the U-Net on the Oxford dataset demonstrates the generalisability of this approach and yielded a mean Dice coefficient of 0.962. Results of the proposed U-Net also compare favourably to *Hamwood *et al. who evaluated a fully convolutional network using the Milwaukee dataset and achieved Dice coefficient of 0.989, true positive rate of 0.987, and false discovery rate of 0.009 on the confocal dataset^19^.

Although the synthetic data provided a realistic cone photoreceptor mosaic that was modelled on histologic data, the images did not simulate rod photoreceptors or blood vessels and therefore were not truly representative of real data, particularly outside the fovea. Another limitation of this study includes the lack of non-confocal AOSLO imaging. Updating the simulation used to produce synthetic data to improve agreement between synthetic and real datasets and to simulate non-confocal AOSLO images will enable future work to improve model accuracy and aid automated rod photoreceptor identification. The effects of noise and residual aberration were added to the synthetic images to represent real confocal AOSLO images of adequate quality for manual cone identification. Acquiring real images of this quality may not always be possible, for example in disease states, and updating the synthetic images used to represent real images of a more variable image quality will enable future work to investigate the effect of image quality on automated cone identification and potential utility in clinical settings. Using real datasets with images from a greater range of retinal locations will enable future work to demonstrate additional generalisability and flexibility of the model.

Supervised deep learning models are trained using labelled data. Poor repeatability and reliability of cone identification^20,21^ from manual marking of training labels will adversely affect a model’s performance. Utilising datasets that have been manually marked by multiple graders to improve agreement may help to improve future performance.

This study demonstrates the utility of using synthetic data to overcome the limitation of confocal AOLSO data availability to develop a deep learning algorithm for the fully automated detection of cone photoreceptors. This approach has added advantages of improving generalisability of the deep learning algorithm to confocal AOSLO images acquired from different instruments and to a larger range of retinal eccentricities, as demonstrated in this study. A similar approach to what is described here will require further study but may be useful for photoreceptor identification in retinal disease. Provided models of photoreceptor degeneration exist, large synthetic datasets could be generated at a fraction of the cost of real data from diseased retinae. A fully automated deep learning algorithm for cone photoreceptor identification in retinal disease could be used to quantitatively analyse the photoreceptor mosaic. This may help to realise the potential for wider use of AOSLO imaging for providing cell-specific imaging biomarkers that can be used for diagnosis, prognosis, understanding disease mechanisms, and evaluating novel treatments.

## Data Availability

The datasets supporting the conclusions of this article are available in the following GitHub repositories, https://github.com/DavidCunefare/CNN-Cone-Detection/tree/master/Images and Results/Confocal as cited by Cunefare et al.^1^ and https://github.com/LauraKateYoung/ERICA as cited by Young et al.^11^.

## Abbreviations

AOSLO: Adaptive optics scanning laser ophthalmoscope
CNN: Convolutional neural network
C-CNN: Confocal CNN
ERICA: Emulated Retinal Image CApture
FN: False negatives
FP: False positives
GTDP: Graph-theory and dynamic programming
nW: Nanowatts
SNR: Signal-to-noise ratio
TP: True positives
